# Effect of food availability on the growth and thermal physiology of juvenile Dungeness crabs (*Metacarcinus magister*)

**DOI:** 10.1093/conphys/cov013

**Published:** 2015-03-31

**Authors:** Katherine M. McLean, Anne E. Todgham

**Affiliations:** 1 Department of Biology, San Francisco State University, San Francisco, CA 94132, USA; 2 Department of Animal Science, University of California, Davis, CA 95616, USA

**Keywords:** Crab, energy allocation, food limitation, thermal physiology

## Abstract

Juvenile Dungeness crabs spend ~1 year in the San Francisco Estuary, where they undergo considerable growth before returning to the coastal ocean. Previous studies suggest that competition, food scarcity and avoidance of conspecifics may cause some juvenile Dungeness crabs in the San Francisco Estuary to become food limited. Food limitation may force these crabs to forage in higher temperature intertidal environments in the estuary, exposing them to stressful conditions in order to sustain growth and, potentially, necessitating physiological trade-offs in energy allocation between growth and stress tolerance. To investigate the effects of food limitation on aerobic metabolism and physiological performance of crabs, we assessed growth, moulting frequency, metabolic rate, citrate synthase and malate dehydrogenase enzyme activity and cardiac performance, as an index of temperature sensitivity and upper temperature tolerance. Summer- and winter-caught crabs were acclimated to either a high- or a low-food ration for 5 weeks. Overall, our results demonstrated that while food limitation had a negative effect on growth of juvenile Dungeness crabs in both the summer and the winter feeding trials, crabs in the low-food group maintained both metabolic rate at ambient San Francisco Estuary temperatures (15°C; summer trial only) and upper temperature tolerance as determined by failure of cardiac function when compared with crabs in the high-food group (summer and winter trials). Therefore, the ability to maintain stress tolerance when food is limited appears to come as a physiological trade-off to growth. Food-limited crabs were unable to increase their metabolic rate to the same level as that achieved by well-fed crabs; therefore, if exposure to elevated temperatures persists and requires more energy than can be met by crabs in their food-limited state, physiological performance may be compromised.

## Abstract (precis)

The San Francisco Estuary is an important nursery ground for juvenile Dungeness crabs and is predicted to experience increasing temperatures with climate change. When food was limited, growth in crabs was reduced and results suggested that crabs may be more sensitive to mean changes in temperature rather than heat waves.

## Introduction

Food limitation can affect energy homeostasis in an organism, necessitating trade-offs in the allocation and use of energy (Bradley *et al.*, 1991; Glazier and Calow, 1992). Aerobic metabolism may shrink to match the reduced energy supply, leading to less energy being available for allocation to various biological processes (Ansell, 1973; Wallace, 1973; Depledge, 1985). The reduction in energy allocation can occur globally, with all physiological processes receiving less ATP, or as trade-offs between physiological processes if energy is preferentially allocated to some processes and diverted away from others (Bradley *et al.*, 1991; Glazier and Calow, 1992). Sokolova *et al*. (2012)propose an integrative and theoretical bioenergetics framework for assessing the physiological trade-offs associated with responding to environmental change in aquatic invertebrates. The framework incorporates both the oxygen- and capacity-limited thermal tolerance hypothesis (Pörtner, 2010) and the dynamic energy budget models proposed by Kooijman (2010)and provides a comprehensive approach to capture the trade-offs associated with maintaining energy balance between basal maintenance and fitness-related functions (termed aerobic scope functions) during stress. When food is limited, an animal may preserve basal maintenance functions (e.g. ion and acid–base regulation, ventilation, circulation), typically the first priority of energy allocation (Sokolova *et al.*, 2012), but sacrifice aerobic scope functions, such as growth, reproduction or activity (Terwilliger and Dumler, 2001; Comoglio *et al.*, 2005; Wen *et al.*, 2006). If food limitation is extreme, the animal may also reduce basal maintenance functions, such as circulation and ventilation, in order to balance the reduced energy supply with reduced energy demand (Ansell, 1973; Depledge, 1985; Naylor and Taylor, 1999). When exposed to an environmental stressor, such as increased temperature, a food-limited animal may lack the energy necessary to mount a stress response, recover from a stressor and maintain homeostasis (Sokolova *et al.*, 2012). Overall, energy imbalance may not only lead to a reduction in stress tolerance but may also scale up to impact long-term population persistence through reduced energy supply to growth and reproduction, key -fitness-related functions (Woiwode and Adelman, 1992; Nyamukondiwa and Terblanche, 2009; Woodman, 2010; Sokolova *et al*., 2012).

Dungeness crabs, *Metacarcinus magister*, are an economically and ecologically important species distributed along the eastern Pacific coast from Alaska to Santa Barbara (CA, USA), with juveniles using the San Francisco Estuary (SFE) as a nursery habitat (Wild and Tasto, 1983). Females carry eggs in the autumn, which hatch as free-swimming zoea larvae into the water column of the coastal ocean from late December to mid-January (Hieb, 1999). Pelagic zoea develop into megalopae, then settle as juveniles and are swept to either coastal or estuarine waters from March to June (Hieb, 1999). Juveniles entering the SFE have been shown to grow twice as quickly as those reared along the coast (Wild and Tasto, 1983; Hieb, 2012). After living in the estuary for ~1 year, juveniles migrate to the coast, where they contribute to an annual $30.4 million California Dungeness crab fishery industry (Larinto *et al.*, 2013). Strong year classes from SFE-reared crabs have been shown to correspond to strong fishing seasons in the California Dungeness crab fishery once the crabs reach legal size (Hieb, 2012).

When crab densities are high in the SFE, competition for food resources, food scarcity and avoidance of conspecifics may necessitate foraging in suboptimal thermal habitats and could lead to some crabs experiencing food limitation (Stevens *et al.*, 1984). In fact, juvenile Dungeness crabs have been shown to migrate from subtidal habitats to shallow intertidal mudflats in pursuit of prey (Stevens *et al.*, 1984). These mudflats are warmer than the subtidal zones (Holsman *et al*., 2003), so crabs are more likely to experience thermal stress when they are foraging for food. Juvenile Dungeness crabs in Willapa Bay (WA, USA), a typical large northeastern Pacific estuary, can occur at densities so high that they must migrate to intertidal zones to forage because the subtidal prey biomass alone cannot meet the energetic demands of the crabs (Holsman *et al.*, 2003). In a study from September to December 1979 in the SFE, juvenile Dungeness crab stomachs were, on average, approximately half-full, and 5% of crabs had completely empty stomachs (Wild and Tasto, 1983). Starved adult Dungeness crabs will spend more time foraging at high temperatures than well-fed crabs, suggesting that crabs in poorer condition will seek stressful environments for food (Curtis and McGaw, 2012). Whether food limitation impacts the physiological sensitivity of juvenile Dungeness crabs to acute thermal stress and upper temperature tolerance is unknown but likely, given the pervasive effects of temperature on crustacean physiology (Whiteley *et al.*, 1997, 2001). Furthermore, food limitation has been shown to affect both the physiological response to stressors (e.g. hypoxia, McGaw, 2005; temperature, McGaw and Whiteley, 2012) and temperature preference in adult Dungeness crabs (Curtis and McGaw, 2012). If food limitation impairs the stress tolerance of juvenile Dungeness crabs then there are important physiological trade-offs to consider if hungry crabs are more likely to enter a stressful environment to forage for food during a critical development stage.

Currently, it is unknown how food limitation affects energy use and allocation in juvenile Dungeness crabs. We evaluated the effects of food limitation on physiological performance and acute thermal tolerance in juvenile Dungeness crabs caught in the summer and winter in order to gain a better understanding of how changes in aerobic metabolism affect energy (i.e. ATP) supply and the energy allocated to basal maintenance compared with aerobic scope functions (Sokolova *et al*., 2012). As Dungeness crabs spend ~1 year in the SFE, it is important to examine the effect of food limitation across seasons to determine whether crabs are more vulnerable during a particular developmental lifestage and/or during a particular time of year. The experiments were designed to answer the following three main questions.

(i) How does food limitation affect juvenile Dungeness crab energy demand as measured by growth (e.g. changes in weight, carapace width and moulting frequency), basal heart rate (i.e. circulation) and aerobic ATP supply, through measures of basal oxygen consumption rates and aerobic capacity of metabolic enzymes?
(ii) How does food limitation affect upper temperature tolerance and temperature sensitivity of juvenile Dungeness crabs, as measured by upper critical thresholds in cardiac function, cardiac performance curves and oxygen consumption rates during thermal stress?
(iii) Does season and/or developmental stage affect how food limitation impacts thermal tolerance and temperature sensitivity of crabs?

Identifying which physiological processes are most negatively affected by food limitation will provide much needed information on the physiological trade-offs and costs associated periods of rapid growth in a highly variable environment in juvenile Dungeness crabs in the nursery grounds of the SFE. Furthermore, the SFE is predicted to experience a 1.4–4.5°C increase in annual mean temperature by the year 2099 (Cayan *et al*., 2006; Brown *et al*., 2013), and the number of days when water temperature will exceed 25°C as well as the number of extreme thermal events are also predicted to increase (Hayhoe *et al*., 2004; Cloern *et al*., 2011; Wagner *et al*., 2011). Therefore, an understanding of the physiological trade-offs associated with food limitation and stress tolerance will be important considerations for predictions of how juvenile Dungeness crabs and the associated fishery will cope with temperature changes predicted to occur by the end of the century.

## Materials and methods

### Animal collection and holding

#### Summer feeding trial

Juvenile Dungeness crabs (*n* = 120, mass = 3.44 ± 0.01 g, carapace width = 26.69 ± 0.03 mm, 75% male) were caught from three locations in the San Rafael Bay (CA, USA) using a 7.6 m beach seine net from 24 July to 2 August 2012 (location 1, 37°96′30″N, 122°48′97″W; location 2, 37°95′19″N, 122°48′83″W; and location 3, 37°94′59″N, 122°48′36″W). Crabs were transported in aerated coolers from collection sites to outdoor tanks at the Romberg Tiburon Center for Environmental Studies, San Francisco State University in Tiburon (CA, USA). There were no crab mortalities during transport.

At the start of the experiment, crabs were placed in individual round containers (1.65 l) for the duration of the feeding trial. This was done to ensure that each crab could be fed a specific amount of food that could be eaten only by that crab. Each individual crab container had three rectangular holes covered with window screening along the sides to allow for adequate water exchange, and crabs were provided with a 7.5 cm piece of 1.9 cm diameter PVC as shelter, which they occupied roughly 50% of the time. Each container was supplied with flow through bay water and bubbled with an air stone. All individual containers were placed in one of six 410 l outdoor circular tanks that received flow through bay water from the San Francisco Bay (17.1 ± 0.1°C, salinity 31.0 ± 0.1). Tanks were exposed to the natural photoperiod (13.5 h light and 10.5 h dark; Online Photoperiod Calculator V 1.94 L) but were covered with shade cloth, preventing 70% of ambient light transmittance to limit sun exposure during the day, because crabs in nature would not experience full sun at depth in the San Francisco Bay. Each outdoor tank contained 20 individual containers and was designated as either a high- or a low-food tank (feeding levels are described under ‘*Feeding acclimation and growth*’). The high- and low-food tanks were replicated three times for a total of six tanks (*n* = 60 crabs per feeding group). Feeding groups were kept in separate tanks because feeding time was not synchronized between high- (fed every 48 h) and low-feed groups (fed every week) and we did not want the low-food crabs to get the scent of food, in the absence of available feeding opportunities.

#### Winter feeding trial

Juvenile Dungeness crabs (*n* = 48, mass = 127.66 ± 7.66 g, -carapace width = 91.28 ± 1.96 mm, 54% male) were caught from three locations in the San Rafael Bay (CA, USA) between 6 and 11 March 2013. Crabs were collected for the winter feeding trial using different methods from the summer trial because older juvenile crabs move to deeper water in the SFE during the winter months. Opera traps were baited with squid and set daily at the Romberg Tiburon Center for Environmental Studies (37°89′03″N, 122°44′63″W). Crabs were also collected from the Central Bay and San Rafael Bay by the California Department of Fish and Wildlife using an otter trawl. Crabs from the California Department of Fish and Wildlife were transported in aerated coolers from the Berkeley Marina to outdoor tanks at the Romberg Tiburon Center for Environmental Studies. There were no crab mortalities during transport.

Crabs in the winter trial were ~7 months older than the crabs in the summer trial and were therefore larger, requiring larger individual containers (7.5 l) for the duration of the feeding trial. Individual containers had mesh-covered holes on the sides to allow for adequate water exchange, a rock for shelter, and were supplied with flow through bay water and an air stone. Similar to the summer trials, these individual containers were held within 410 l outdoor circular tanks that received flow through bay water from the San Francisco Bay (13.8 ± 0.2°C, salinity 28.3 ± 0.3) and were exposed to the natural photoperiod (12.5 h light and 11.5 h dark) but were covered with shade cloth. Each large circular tank contained eight individual containers and was designated as either a high- or a low-food tank (feeding levels are described under ‘*Feeding acclimation and growth*’). The high- and the low-food tanks were replicated three times for a total of six tanks (*n* = 24 crabs per feeding group).

### Feeding acclimation and growth

#### Summer feeding trial

Crabs were acclimated to two feeding regimens for 36 days. Crabs in the high-food group were fed ~9% of their body weight in frozen squid tissue every 48 h, while crabs in the low-food group were fed ~1.5% of their body weight in frozen squid tissue once per week. Feeding levels were determined using information from the published literature (Asson-Batres, 1986; Curtis and McGaw, 2010) as well as a preliminary feeding trial conducted in summer 2012 (data not shown). It was observed that after 24 h there was no food left in containers of either group. Squid was purchased frozen at a local grocery store and prepared by thawing the tissue, blotting it dry, weighing the appropriate amount and placing it in an ice-cube tray filled with bay water. The tray was then frozen, and crabs were fed by dropping an ice cube into each individual container during each feeding period.

During the feeding trial, the water temperature, salinity and percentage of oxygen were measured daily in five individual crab containers per large outdoor circular tank and in the surrounding water of each tank. This ensured that water quality measurements were made in all of the 120 individual crab containers weekly. The average temperature for the individual containers in the six large outdoor tanks for the summer feeding trial was 17.1 ± 0.1°C, average salinity was 31.0 ± 0.1, and average oxygen content was 91.35 ± 0.55%. Containers were checked daily for survival and crab moults, which were removed, weighed and measured. Additionally, each crab was weighed and its carapace width, just before the 10th spine, measured once per week. After the feeding trial, measures of physiological performance were assessed over subsequent days and, as a result, the feeding ration was continued until all performance measures were complete (the total duration of the experiment was 50 days).

#### Winter feeding trial

Crabs were fed the same feed rations as those in the summer trial during the 39 day winter feeding trial (the total duration of the experiment was 47 days when considering physiological performance assessments). The average temperature for the individual containers in all six tanks for the winter feeding trial was 13.8 ± 0.2°C, average salinity was 28.3 ± 0.3, and average oxygen content was 93.26 ± 0.67%.

#### Analysis of growth, moulting and survival

Differences in weight and carapace width (CW) were calculated after the feeding trial in all the crabs and grouped as those that had moulted and those that had not. Moulting was assessed daily and monitored until the end of the experiment when all the physiological performance measures were concluded (i.e. 50 days in the summer feeding trial and 47 days in the winter feeding trial). Growth increment was calculated by dividing the difference in pre- and post-moult CW by pre-moult CW as follows: [(post-moult CW – pre-moult CW)/-pre-moult CW] × 100 (Tarling *et al.*, 2006; Kobayashi, 2012).

### Metabolic rate during acute temperature exposure

Oxygen consumption trials were performed only in the crabs in the summer feeding trial because insufficient numbers of Dungeness crabs were collected in the winter to carry out both cardiac performance trials and measurements of oxygen consumption rate. Twenty-four hours before oxygen consumption trials, crabs were placed in open 240 ml glass respirometers (Wheaton, Millville, NJ, USA) and were not fed during this period. Respirometers were covered with window screening to prevent escape and supplied with flow through bay water. Oxygen consumption following acute temperature exposure was measured in closed respirometers at four temperatures (15, 20, 25 and 30°C) in crabs (*n* = 8) from both feeding groups (four crabs per group per day for 8 days). Atmospheric pressure was recorded from the San Francisco Bay Environmental Assessment and Monitoring Station north of the Tiburon Peninsula (37°53′20″N, 122°26′48″W) and retrieved daily from the website http://sfbeams.sfsu.edu/.

Immediately before measurement, the crab was gently transferred to a second respirometer filled with water at the target temperature, either ambient temperature (handling control) or an elevated temperature, by allowing the crab to move between glass jars. Crabs were then allowed to recover from the transfer to the experimental temperature with the caps removed from the respirometer for 30 min. The respirometers were then capped underwater at their experimental temperatures to ensure that no air bubbles were left in the respirometer. The respirometer was then placed in a temperature--controlled aluminum block maintained at the target temperature for the duration of the metabolic trial. The block was placed on a stir plate, and water in the respirometer was mixed using a stirring apparatus above the crab. The respirometer was covered with a piece of black plastic to minimize disturbance to the crab. Oxygen consumption was recorded using Presens Fibox optical sensor spots (accuracy of ±0.4% at 20.9% O_2_) and an oxygen meter (Presens, Regensburg, Germany) that measured changes in the percentage air saturation continuously until the water reached 70% air saturation, as described by Bjelde and Todgham (2013). Following each respirometry trial, crab wet weight was recorded. The rate of oxygen consumption was calculated for each trial over the period when air saturation fell from 100 to 80%. Oxygen consumption in a respirometer containing only bay water was subtracted from the oxygen consumption rate of each crab to account for microbial activity in the bay water. Mass-specific metabolic rate (${\dot{M}}_{O_{2}}$) was then calculated in micromoles of O_2_ per hour per gram of wet weight.

### Citrate synthase assay

Citrate synthase (CS) is a rate-limiting enzyme in the citric acid cycle and an indicator of aerobic metabolic capacity (Berges *et al.*, 1993). Citrate synthase specific activity of gill tissue, a metabolically active tissue essential for oxygen uptake and therefore energy supply, was measured at 25°C following a protocol modified from Bergmeyer and Karlfried (1978). Citrate synthase activity was measured at the end of the summer feeding trial in both the low- (*n* = 6) and the high-food group (*n* = 8). In the winter feeding trial, CS specific activity was measured before the start of the feeding trial in addition to the end of the feeding trial in both low- and high-food groups (*n* = 8 for all groups). Briefly, frozen gill tissue was homogenized in 200 μl of 50 mM imidazole hydrochloride buffer (pH 8.2 at 20°C) using a hand-held homogenizer (Pro200; ProScientific, Oxford, CT, USA). The homogenate was then centrifuged at 13 000***g*** for 8 min at 4°C. A 1:10 dilution was made in 50 mM imidazole hydrochloride buffer. Diluted homogenate (10 μl) was loaded onto a 96-well plate (Costar; Corning, Tewksbury, MA, USA) and assayed in 200 μl of 50 mM imidazole hydrochloride buffer (pH 8.2 at 20°C), 0.1 mM 5.5′-dithio-bis-2-nitrobenzoic acid, 0.15 mM MgCl_2_ and 0.012 mM acetyl-CoA (lithium salt). Oxaloacetate (40 mM) was added to one set of triplicates and omitted from another set to serve as blanks. The plate was shaken at a slow speed for 15 s, then read at 412 nm every 44 s for 2 h using a Synergy HT microplate reader (BioTek, Winooski, VT, USA). The concentration of protein in the homogenate was measured using the bicinchoninic acid method (Smith *et al.*, 1985). Citrate synthase specific activity (in micromoles of oxaloacetate oxidized per minute) is expressed as international units (IU) per gram of protein.

### Malate dehydrogenase assay

Malate dehydrogenase (MDH) is an enzyme involved in -several metabolic pathways, including the citric acid cycle and the malate–aspartate shuttle, in which mitochondrial MDH converts oxaloacetate to malate and cytosolic MDH converts malate back to oxaloacetate (Berg *et al.*, 2002; Minarik *et al.*, 2002). Malate dehydrogenase specific activity, like CS specific activity, is an indicator of aerobic metabolism; however, because MDH is involved in other metabolic pathways, measuring both CS and MDH specific activity can provide a more comprehensive picture of metabolic activity. The MDH specific activity in gill tissue was measured at 25°C following a protocol modified from Bergmeyer and Karlfried (1978). Malate dehydrogenase activity was measured at the end of the summer feeding trial in both the low- (*n* = 6) and the high-food groups (*n* = 8). In the winter feeding trial, MDH specific activity was measured before the start and at the end of the feeding trial in both low- and high-food groups (*n* = 8 for all groups). Briefly, frozen gill tissue was homogenized in 200 μl of 50 mM potassium phosphate buffer (pH 6.8 at 20°C). The homogenate was centrifuged for 8 min at 13 000***g*** at 4°C. A 1:10 dilution was made in 50 mM potassium phosphate buffer. Diluted homogenate (5 μl) was loaded onto a 96-well plate in triplicates and assayed in 200 μl of 50 mM potassium phosphate buffer, 0.2 mM oxaloacetate and 0.16 mM NADH. Potassium phosphate buffer (5 μl) was loaded onto the plate as a blank. The plate was shaken at a slow speed for 10 s and then read at 340 nm every 27 s for 2 h. The concentration of protein in the homogenate was measured using the bicinchoninic acid method. The MDH specific activity (in micromoles of oxaloacetate oxidized per minute) is expressed as international units per gram of protein.

### Cardiac performance during ramped increases in temperature

#### Measurements of heart rate

Dungeness crab heart rates were measured at the end of both the summer (*n* = 10 per feeding group) and winter feeding -trials (*n* = 8 per group) using methodology from Stillman (2003), modified for larger crabs, to assess temperature sensitivity and upper critical thermal limits of cardiac performance. Food was withheld for 24 h before heart rate experiments. Two small holes were made into the carapace on either side of the heart using an 18-gauge hypodermic needle. Copper electrodes were inserted into each hole. For the smaller crabs from the summer feeding trial, a small piece of cork was affixed to the carapace of each crab using an insta-cure ethyl cyanoacrylate adhesive (Bob Smith Industries, Atascadero, CA, USA). The crabs were then held in place by clipping the cork into a clothes pin that was attached to a plastic frame. The entire frame was lowered into a water bath containing bay water at 12°C, salinity 31.6 ± 0.2. For the larger crabs in the winter feeding trial, crabs were placed in plastic containers with three rectangular holes covered with mesh and lowered into bay water at 12°C, salinity 28.9 ± 0.8. A water bath (Proline RP855; LAUDA-Brinkmann, LP, Delran, NJ, USA) held the water at 12°C for 15 min, representing a low bay water temperature, to account for handling and placement of electrodes. The water temperature was then increased to 36°C at a rate of 6°C h^−1^. For the duration of the heart rate trial, the cardiac performance of each crab was recorded as impedance (a measure of the resistance of a circuit to current when voltage is applied). Impedance converters (UFI model 2991, Morro Bay, CA, USA) were used to convert heart recordings to voltage signals using the PowerLab Chart 5 program (ADInstruments, Colorado Springs, CO, USA), and heart rate (in beats per minute) was calculated from the heart rate traces. Crab hearts beat continuously throughout the trial, with limited, short-duration (<5 s) pauses, and heart rate was therefore calculated during long periods when the heart was beating.

#### Analysis of cardiac performance

To assess the temperature sensitivity of the cardiac function of crabs from different feeding trials and across different seasons, performance curves of heart rate were generated for each crab at 3°C temperature intervals from 12 to 27°C and then at 1°C intervals from 29 to 35°C during the ramping protocol (Bjelde and Todgham, 2013). Recordings of heart rate 30 s in duration were captured at 30 s intervals for the duration of a particular temperature ±0.5°C (i.e. for 24°C, heart rate was taken from recordings between 23.5 and 24.5°C), and individual mean heart rate was then calculated from all the data points acquired for a single temperature.

#### Break-point and flat-line temperatures

To assess the upper critical temperature tolerance of cardiac function, the final break-point temperature (BPT), the temperature at which a crab's heart rate begins to decline rapidly and not recover, was measured by plotting heart rate (in beats per minute) against temperature and finding the inflection of the plot (sharp decrease in slope following a peak in heart rate; Stillman, 2003; Bjelde and Todgham, 2013). Two best-fit regression lines were drawn over the pre-BPT data (ascending) and the post-BPT data (descending). The intersection of the two lines was found as described by Nickerson *et al.* (1989)using R (R Development Core Team, 2008) and was used to determine the final BPT. The flat-line temperature (FLT), the temperature at which all cardiac function ceased, was measured as the temperature with the last heart beat for each crab.

### Statistical analyses

Statistical analyses were conducted in SPSS (IBM Corporation, Armonk, NY, USA). Data were first tested for normality (Shapiro–Wilks test) and homogeneity of variance (Levene's test) to ensure that parametric analysis assumptions were met. If data failed to meet assumptions of normality and homogeneity of variance, then either a Mann–Whitney *U* test was performed to compare two groups or a Kruskal–Wallis test was performed to compare three or more groups, followed by *post hoc* Mann–Whitney *U* tests. Bonferroni-corrected α-values were applied for all non-parametric tests to account for multiple comparisons. Survival between crabs in the high- and low-food groups was compared using a X^2^ test, and X^2^ tests were also used to identify the effect of feeding level on the total number of crabs to moult during the feeding trial. All data are presented as means ± SEM, unless noted.

## Results

### Growth

#### Summer feeding trial

Fifty-eight of the 60 crabs (96.67%) in the high-food group and 52 of the 60 crabs (86.67%) in the low-food group survived the feeding trial (data not shown). There was no significant difference in survival between crabs in the high- and low-food groups after the 36 day feeding trial (X^2^ = 2.727, *P* = 0.099).

**Figure 1:**
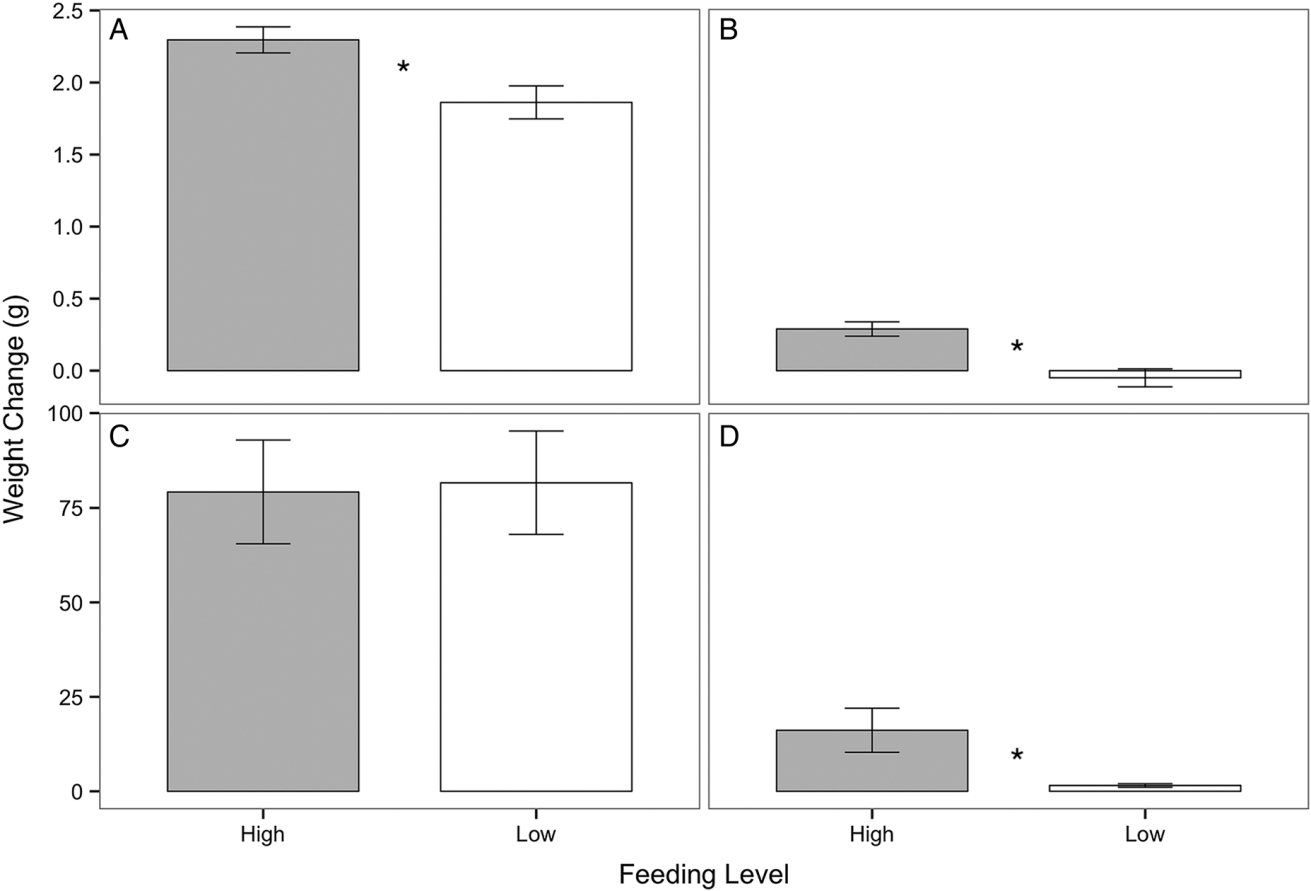
Change in wet weight (in grams) of *Metacarcinus magister* from high- (grey) and low-food groups (white) for the summer feeding trial when (**A**) moulted crabs (*n* = 43 high-food group, *n* = 25 low-food group) and (**B**) non-moulted crabs (*n* = 15 high-food group, *n* = 30 low-food group) were considered separately, and for the winter feeding trial when (**C**) moulted crabs (*n* = 9 high-food group, *n* = 7 low-food group) and (**D**) non-moulted crabs (*n* = 14 high-food group, *n* = 16 low-food group) were considered separately. Data are presented as means ± SEM. Asterisks represent significant differences in wet weight between high- and low-food groups within a particular comparison (Mann–Whitney *U* test, α = 0.017).

Crabs in the high-food group had a significantly greater increase in wet weight compared with crabs in the low-food group (Mann–Whitney *U* = 685.5, *P* = 0.0001, two tailed, α = 0.017 with Bonferroni correction) over the course of the feeding trial. When only moulted crabs were considered, crabs the high-food group had a significantly greater increase in weight than crabs in the low-food group (Fig. 1A; Mann–Whitney *U* = 292.0, *P* = 0.002, two tailed, α = 0.017). When only non-moulted crabs were considered, crabs in the high-food group gained significantly more weight than crabs in the low-food group, which lost weight on average (Fig. 1B; Mann–Whitney *U* = 42.5, *P* = 0.0001, two tailed, α = 0.017). Only moulted crabs were considered when analysing changes in CW, because non-moulted crabs were not expected to change in size. There was no significant difference in CW change between crabs in the high- (5.80 ± 0.12 mm) and low-food group (5.36 ± 0.22 mm) that moulted during the summer feeding trial (Mann–Whitney *U* = 400.5, *P* = 0.077, two tailed, α = 0.017 with Bonferroni correction, data not shown).

**Figure 2:**
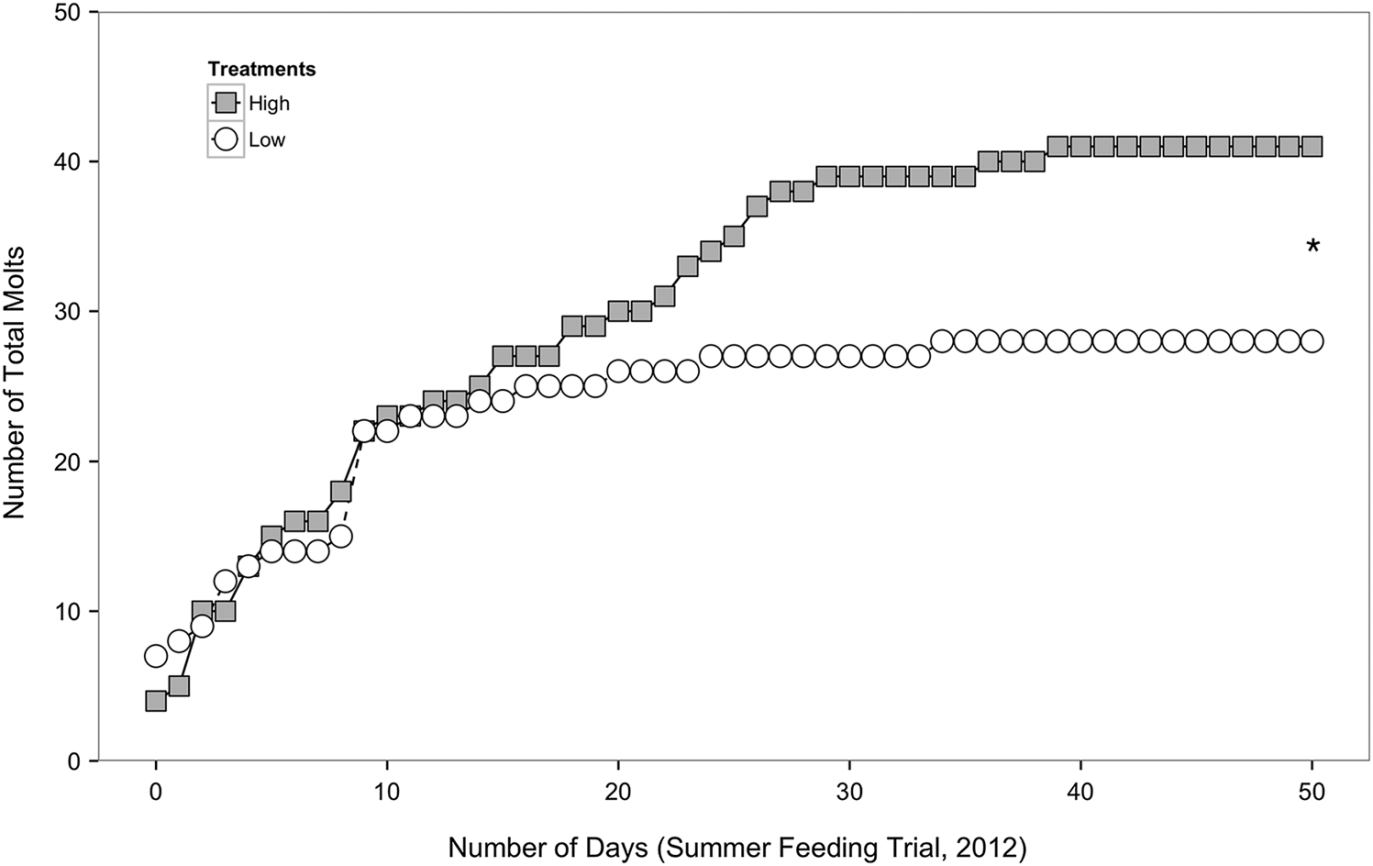
Total number of *M. magister* crabs that moulted over the course of the summer feeding trial from the high- (grey, *n* = 41) and low-food groups (white, *n* = 28). An asterisk represents a significant difference in the total number of crabs moulted by the end of the experiment.

The total number of crabs in the high-food group that moulted by the end of the feeding trial (*n* = 41) was significantly higher than the number of crabs in the low-food group that moulted (*n* = 28; X^2^ = 4.910, *P* = 0.027). Crabs in the high-food group moulted at a nearly constant rate (approximately two crabs per day) for the duration of the feeding trial, whereas crabs in the low-food group moulted at the same pace as crabs in the high-food group for the first 17 days of the experiment (approximately two crabs per day) but the rate of moulting slowed during the final 33 days (approximately one crab every 5 days; Fig. 2).

**Figure 3:**
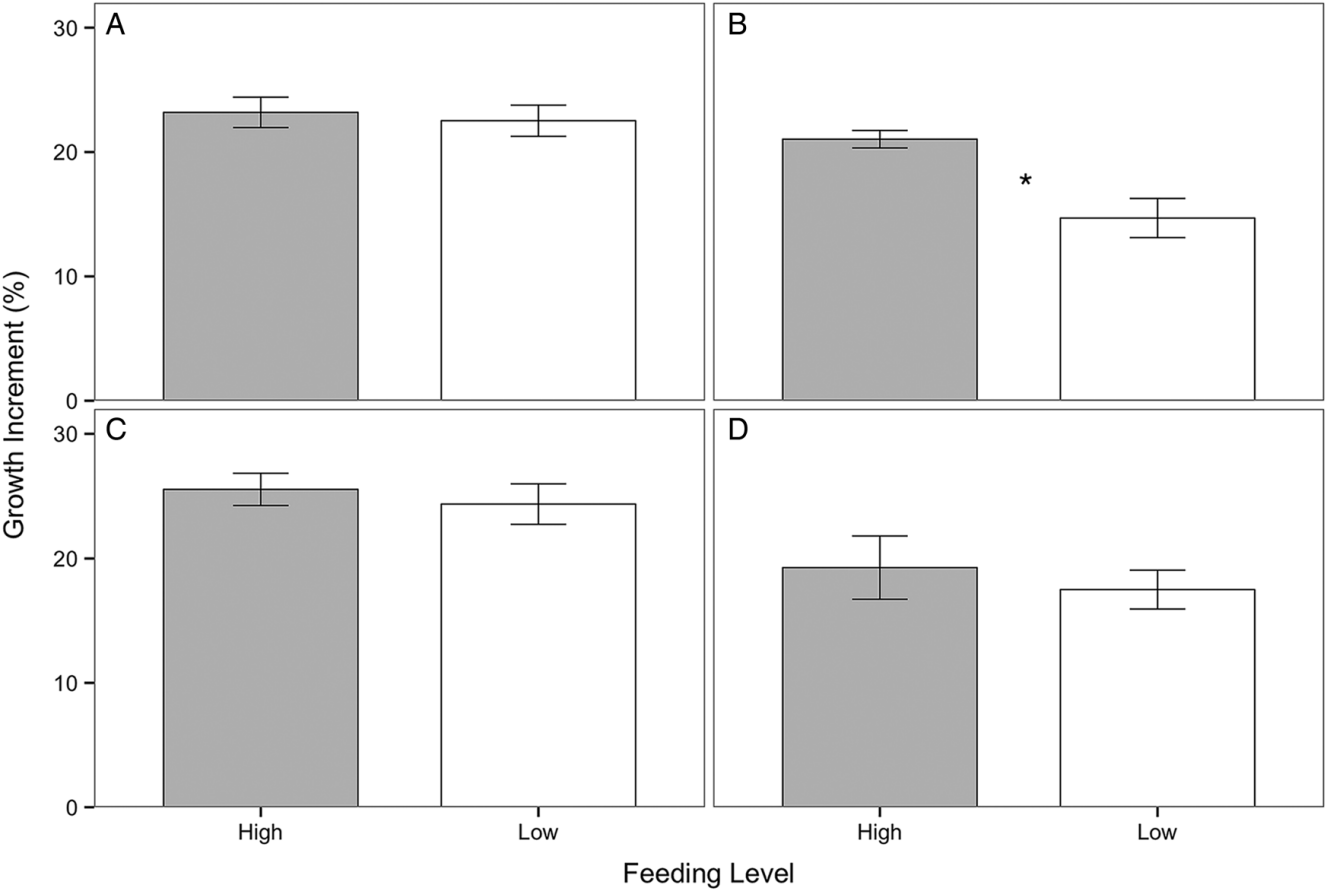
Growth increment (expressed as a percentage) of *M. magister*, calculated as the change in carapace width immediately after a moulting event, for the summer (**A** and **B**) and winter feeding trials (**C** and **D**). For the summer feeding trial, the growth increment is divided into two sections: (A) the first 17 days of the feeding trial, when crabs in the high- (grey, *n* = 26 moulted crabs) and low-food groups (white, *n* = 24) moulted at roughly the same rate; and (B) the final 33 days, when moulting in crabs from the low-food group (white, *n* = 4) was reduced compared with crabs in the high-food group (grey, *n* = 15). For comparisons with the summer feeding trial over the same time periods, the growth increment in the winter feeding trial is also divided into two sections: (C) the first 17 days of the feeding trial (high-food group *n* = 5, low-food group *n* = 3); and (D) the final 30 days of the feeding trial (high-food group *n* = 5, low-food group *n* = 4). Data are presented as means ± SEM. An asterisk represents a significant difference in growth increment between high- and low-food groups for a particular period of time within a given feeding trial (Mann–Whitney *U* test, α = 0.025).

**Figure 4:**
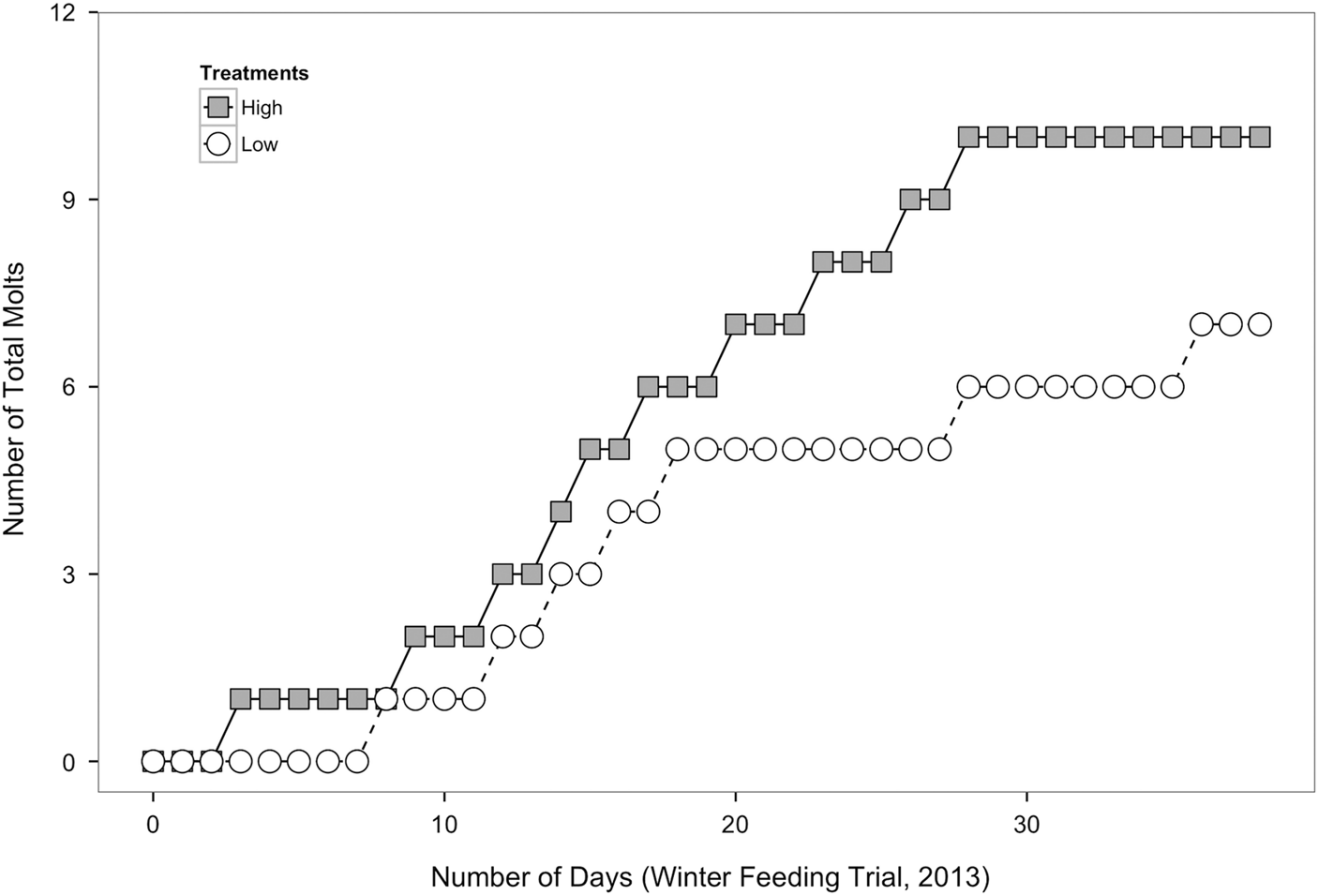
Total number of *M. magister* crabs from the high- (grey squares, *n* = 9) and low-food groups (white circles, *n* = 7) that moulted over the course of the winter feeding trial. One crab from the high-food group moulted twice during the winter feeding trial, so the total number of moults from the high-food group was 10, while the number of crabs that moulted was nine.

For both summer and winter feeding trials, the growth increment (the change in carapace width with moulting) was separated into two time intervals (Fig. 3) based on comparable trends in moulting frequency between feeding groups during both seasons (illustrated in Figs 2and 4). The first segment contained data from the first 17 days of the feeding trial, when crabs in the high- and low-food groups moulted at roughly the same rate. The second segment contained data from the final 33 days (for the summer trial) and 30 days (for the winter trial) of the feeding trial, when crabs in the low-food group appeared to moult at a slower rate than crabs in the high-food group. During the first 17 days of the summer feeding trial, there was no significant difference in growth increment between crabs in the high- and low-food groups (Fig. 3A; Mann–Whitney *U* = 271.5, *P* = 0.431, two tailed, α = 0.025 with Bonferroni correction). There was, however, a significantly higher growth increment for crabs in the high-food group compared with crabs in the low-food group during the final 33 days of the feeding trial (Fig. 3B; Mann–Whitney *U* = 3.0, *P* = 0.007, two tailed, α = 0.025).

#### Winter feeding trial

There were no differences in survival between crabs in the high- (*n* = 23 out of 24, 95.8% survival) and low-food groups (*n* = 23 out of 24, 95.8% survival; X^2^ = 0.043, *P* = 0.836) tested in the winter (data not shown). Crabs in the high-food group had a significantly greater increase in wet weight compared with crabs in the low-food group (Mann–Whitney *U* = 146, *P* = 0.009, two tailed, α = 0.017 with Bonferroni correction) over the course of the feeding trial. When only moulted crabs were considered, there was no difference in weight change between crabs in the high- and low-food groups (Fig. 1C; Mann-Whitney *U* = 34.0, *P* = 0.791, two tailed, α = 0.017). When only non-moulted crabs were considered, crabs in the high-food group gained significantly more weight than crabs in the low-food group (Fig. 1D; Mann–Whitney *U* = 19.0, *P* = 0.0001, two tailed, α = 0.017). The variance in the weight change of non-moulted crabs from both the high- and low-food groups was very low compared with the variance in the weight change of moulted crabs from both the high- and low-food groups. There was no difference in CW change between crabs in the high- (15.67 ± 2.68 mm) and low-food groups (17.79 ± 1.59 mm) that had moulted during the feeding trial (Mann–Whitney *U* = 32.0, *P* = 0.958, two tailed, α = 0.017 with Bonferroni correction, data not shown).

Winter crabs in the high-food group moulted at a nearly constant rate (approximately one crab every 4 days) until the final week of the feeding trial. Crabs in the low-food group appeared to moult at a slower rate compared with crabs in the high-food group (approximately one crab every 5 days; Fig. 4), but there was no significant difference in the total number of crabs that moulted between the high- and low-food groups (X^2^ = 0.364, *P* = 0.546). In order to remain consistent with the growth analysis from the summer feeding trial, growth increment data were separated into two time intervals. During the first 17 days of the feeding trial, there was no significant difference in growth increment between crabs in the high- and low-food groups (Fig. 4; -Mann–Whitney *U* = 9.0, *P* = 0.655, two tailed, α = 0.025 with Bonferroni correction). When the final 30 days of the feeding trial were considered, there was also no significant difference in growth increment between crabs in the high- and low-food groups (Fig. 4; Mann–Whitney *U* = 13.0, *P* = 0.46, two-tailed, α = 0.025).

### Oxygen consumption

**Figure 5:**
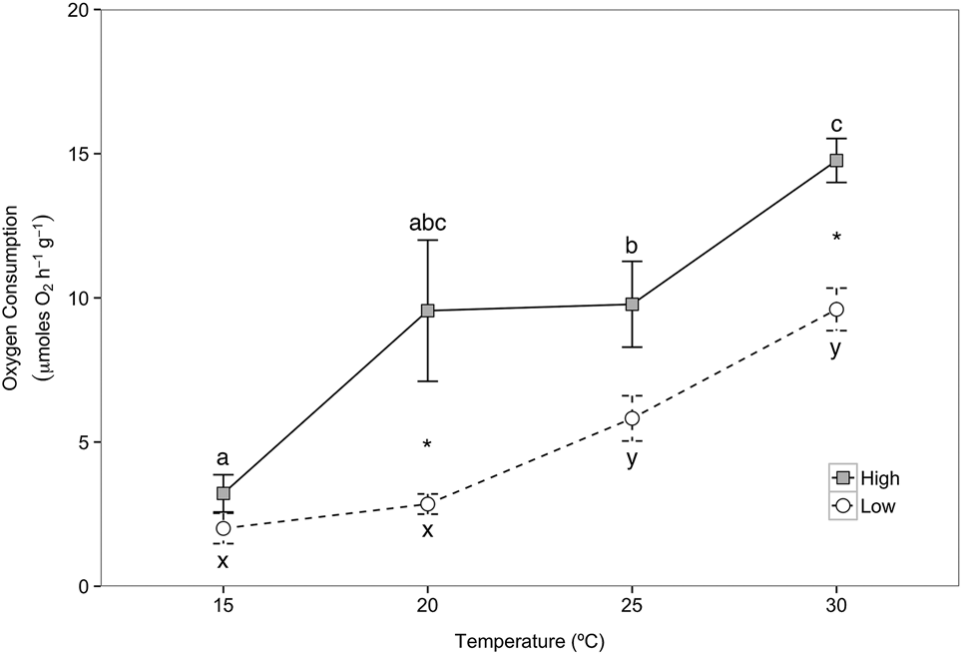
Oxygen consumption (in micromoles of O_2_ per hour per gram of wet weight) of *M. magister* from the high- (*n* = 8) and low-food groups (*n* = 7–8) subjected to acute temperature exposures of 15, 20, 25 and 30°C for 2 h. Data are presented as means ± SEM. An asterisk represents a significant difference in oxygen consumption rate at a particular temperature between high- and low-food groups (Mann–Whitney *U* test, α = 0.013). Within the high- and low-food groups, different letters indicate statistically different rates of oxygen consumption between exposure temperatures (Kruskal–Wallis test, α = 0.008).

Mass-specific metabolic rates measured by oxygen consumption during acute temperature exposures were measured in crabs from the summer feeding trial only. Oxygen consumption rates increased with increasing temperature in crabs from both the high- and low-food groups. At all temperatures above 15°C, crabs in the high-food group had higher ${\dot{M}}_{O_{2}}$ compared with crabs in the low-food group. There were significantly higher values of ${\dot{M}}_{O_{2}}$ for crabs in the high-food groups at 20°C (Mann–Whitney *U* = 6.0, *P* = 0.009, two tailed, α = 0.013 with Bonferroni correction) and 30°C (Mann–Whitney *U* = 3.0, *P* = 0.002, two tailed, α = 0.013) compared with crabs at those temperatures in the low-food group (Fig. 5). For the high-food group, crabs exposed to 25 and 30°C had significantly higher values of ${\dot{M}}_{O_{2}}$ compared with crabs exposed to 15°C (Mann–Whitney *U* = 4, *P* = 0.002, two tailed α = 0.008 with Bonferroni correction for 25°C; and Mann–Whitney *U* = 0, *P* = 0.000, two tailed, α = 0.008 for 30°C). For the low-food group, crabs exposed to 25 and 30°C had significantly higher rates of oxygen consumption -compared with crabs exposed to 15 and 20°C (Mann–Whitney *U* = 2, *P* = 0.001, two tailed, α = 0.008 for 15 compared with 25°C; Mann–Whitney *U* = 0, *P* = 0.001, two tailed, α = 0.008 for 15 compared with 30°C; Mann–Whitney *U* = 3, *P* = 0.002, two tailed, α = 0.008 for 20 compared with 25°C; and Mann–Whitney *U* = 0, *P* = 0.001, two tailed, α = 0.008 for 20 -compared with 30°C).

### Citrate synthase specific activity of gill tissue

**Figure 6:**
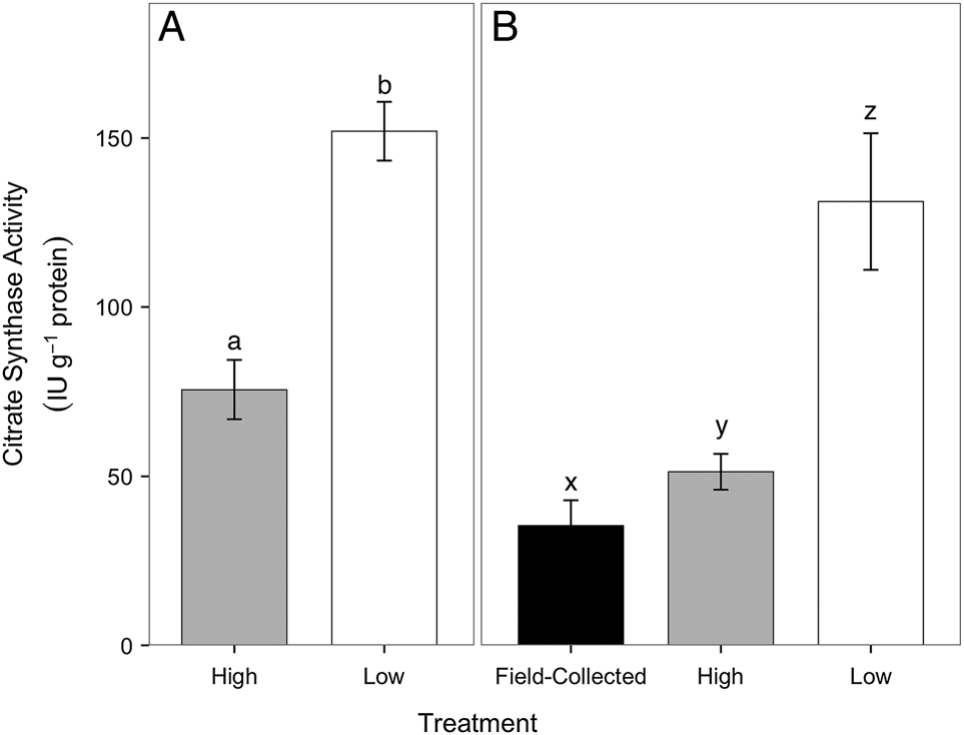
Citrate synthase specific activity (in international units per gram of protein) in gill tissues of crabs from the high- (grey) and low-food groups (white) and field-collected crabs (black) after the summer (**A**) and winter feeding trials (**B**). There were *n* = 8 crabs from the high-food group in both the summer and the winter feeding trials. There were *n* = 7 crabs from the low-food group in the summer feeding trial and *n* = 8 crabs from the low-food group in the winter feeding trial. There were *n* = 8 field-collected crabs. Data are presented as means ± SEM. Different letters represent differences in citrate synthase specific activity within panels (Mann–Whitney *U* test for panel A, Kruskal–Wallis test for panel B, α = 0.017).

In the summer feeding trial, crabs in the low-food group had significantly higher branchial CS specific activity compared with crabs in the high-food group (Fig. 6A; Mann–Whitney *U* = 12.0, *P* < 0.0001, two tailed, α = 0.017 with Bonferroni correction). Likewise, in the winter feeding trial, crabs in the low-food group had significantly higher branchial CS specific activity compared with crabs in the high-food group (Fig. 6B; Mann–Whitney *U* = 36, *P* = 0.001, two tailed, α = 0.017) and compared with field-collected crabs measured at the beginning of the feeding trial (Mann–Whitney *U* = 238, *P* < 0.0001, α = 0.017). Crabs in the high-food group had significantly higher branchial CS activity compared with field-collected crabs measured at the beginning of the feeding trial (Mann–Whitney *U* = 196, *P* = 0.011, α = 0.017). Crabs from the summer feeding trial had significantly higher (~1.5-fold) branchial CS specific activity compared with crabs from the winter feeding trial when food groups were pooled (Mann–Whitney *U* = 350.0, *P* = 0.001, two tailed, α = 0.017).

### Malate dehydrogenase specific activity of gill tissue

**Figure 7:**
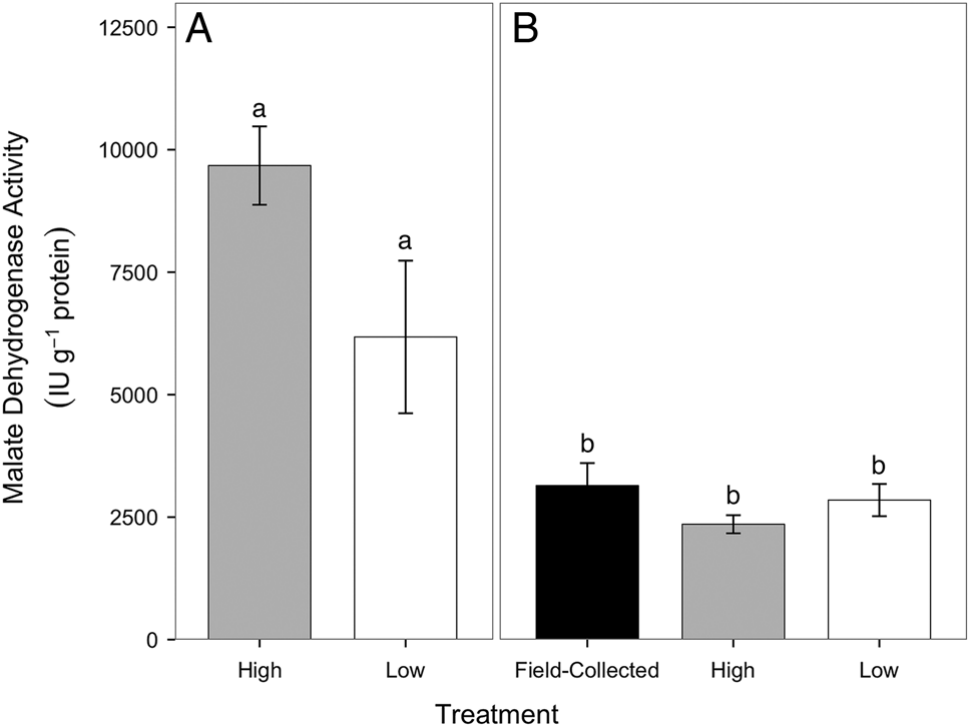
Malate dehydrogenase specific activity (in international units per gram of protein) in gill tissues of crabs from the high- (grey) and low-food groups (white) and field-collected crabs (black) following the summer (**A**) and winter feeding trials (**B**). There were *n* = 8 crabs from the high-food group in both the summer and the winter feeding trials. There were *n* = 7 crabs from the low-food group in the summer feeding trial and *n* = 8 crabs from the low-food group in the winter feeding trial. There were *n* = 8 field-collected crabs. Data are presented as means ± SEM. Different letters represent differences in malate dehydrogenase specific activity within panels (Mann–Whitney *U* test for panel A, Kruskal–Wallis test for panel B, α = 0.017).

In the summer feeding trial, there was no significant difference in branchial MDH between crabs from the high- and low-food groups (Fig. 7A; Mann–Whitney *U* = 145.0, *P* = 0.023, two-tailed, α = 0.017 with Bonferroni correction). In the winter feeding trial, there were no significant differences in branchial MDH specific activity between field-collected crabs measured at the beginning of the experiment, crabs in the low-food group or crabs in the high-food group (Fig. 7B; Kruskal–Wallis, *H* = 2.22, *P* = 0.330, α = 0.017). Crabs from the summer feeding trial had 3-fold higher MDH specific activity compared with crabs from the winter feeding trial when food groups were pooled (Mann–Whitney *U* = 195.0, *P* = 0.000, two tailed, α = 0.017).

### Cardiac performance

#### Cardiac performance curve

**Figure 8:**
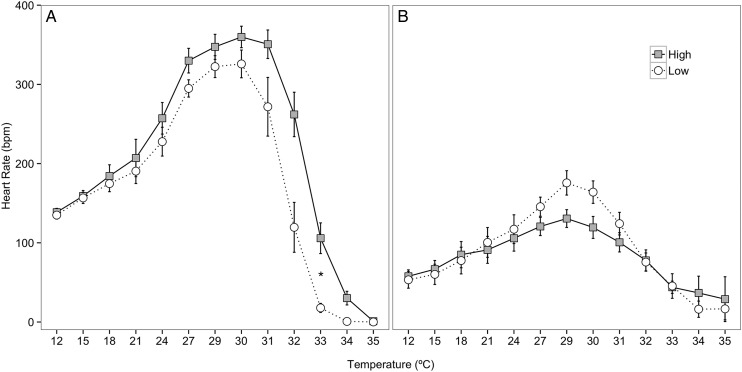
*Metacarcinus magister* cardiac performance curves for crabs in the high- (grey squares) and low-food groups (white circles) in response to increasing temperatures (6°C h^−1^). Heart rates of individual crabs were found by averaging heart rate data from ±0.5°C each temperature interval. Data were then averaged to find the mean heart rate (in beats per minute) ± SEM at each temperature for the summer (**A**; *n* = 8 crabs from the high- and *n* = 9 crabs from the low-food groups) and the winter feeding trials (**B**; *n* = 7 crabs from the high- and *n* = 7 crabs from the low-food groups). An asterisk represents a significant difference in heart rate at a particular temperature between high- and low-food groups (Mann-Whitney *U* test, α = 0.004).

During the summer feeding trial, heart rates in crabs from the high-food group were greater than in crabs from the low-food group at temperatures above 24°C, but the only statistically significant difference was detected at 33°C (Mann–Whitney *U* = 3.0, *P* = 0.001, two tailed, α = 0.004 with Bonferroni correction; Fig. 8A). For the winter feeding trial, there were no statistical differences in heart rate between crabs in the high- and low-food groups at any temperature, although crabs in the low-food group displayed higher heart rates at 29 and 30°C (Fig. 8B). There were no differences in maximal heart rate between crabs in the high- (371.98 ± 12.86 beats min^−1^) and low-food groups (343.51 ± 11.51 beats min^−1^) in the summer feeding trial (Mann–Whitney *U* = 21, *P* = 0.162, two tailed, α = 0.017 with Bonferroni correction, data not shown). There were also no differences in maximal heart rate between crabs in the high- (147.42 ± 8.64 beats min^−1^) and low-food groups (178.78 ± 15.54 beats min^−1^) in the winter feeding trial (Mann–Whitney *U* = 12, *P* = 0.126, two tailed, *α* = 0.017, data not shown). Overall, the maximal heart rate of crabs in the high- and low-food groups in the summer feeding trial, when combined (341.87 ± 11.71 beats min^−1^, *n* = 17), was significantly higher and more than double that of all the crabs in the winter feeding trial (153.14 ± 11.08 beats min^−1^, *n* = 14; Mann–Whitney *U* = 31, *P* = 0.0001, two tailed, α = 0.05, data not shown).

#### Break-point and flat-line temperatures

**Figure 9:**
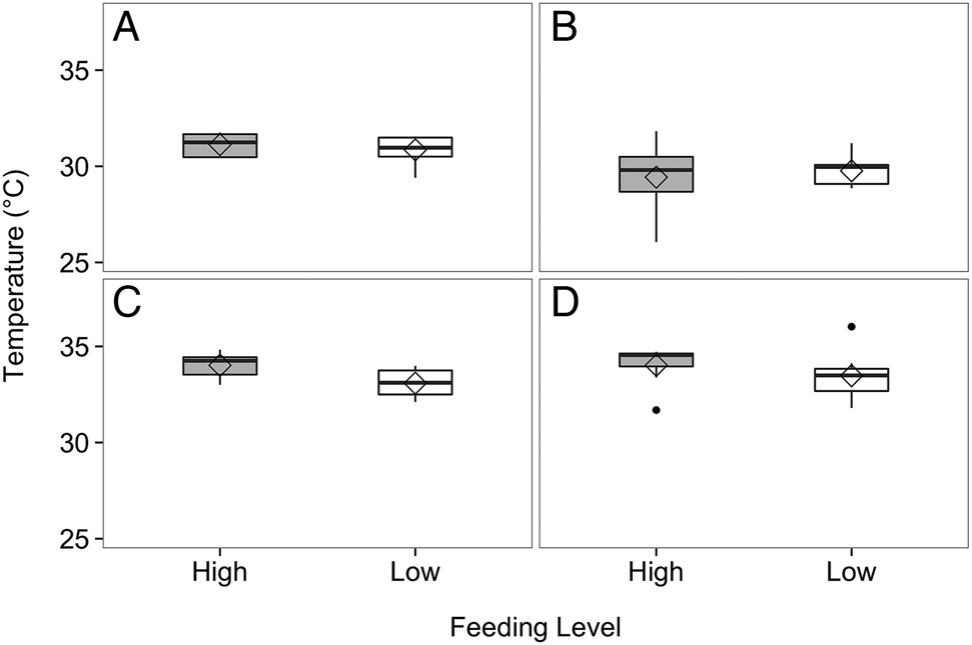
Final break-point temperature (**A**) and flat-line temperature (**C**) in heart rate of crabs in the high- (grey, *n* = 8) and low-food groups (white, *n* = 9) in the summer feeding trial. Final break-point temperature (**B**) and flat-line temperature (**D**) in heart rate of crabs in the high- (grey, *n* = 7) and low-food groups (white, *n* = 7) in the winter feeding trial. The line on the boxplots represents the median, the diamond represents the mean, the box represents the interquartile range, and the whiskers extend to 1.5 times the interquartile range. Points beyond the whiskers are outliers. There were no significant differences in break-point or flat-line temperatures between summer and winter feeding trials (Mann–Whitney *U* test, α = 0.013).

There was no significant difference in upper critical thermal limits of cardiac performance, as determined by final BPT, between crabs in the high- and low-food groups after the 5 week feeding trial (Fig. 9A; Mann–Whitney *U* = 26.0, *P* = 0.336, two tailed, α = 0.0125 with Bonferroni correction). There was also no significant difference in the upper temperature at which heart function ceased (i.e. FLT) between crabs in the high- and low-food groups (Fig. 9C; Mann–Whitney *U* = 12, *P* = 0.021, two tailed, α = 0.0125).

Likewise, in the winter feeding trial there was no significant difference in final BPT between crabs in the high- and -low-food groups (Fig. 9B; Mann–Whitney *U* = 27, *P* = 0.795, α = 0.013 with Bonferroni correction). There was no significant difference in FLT between crabs in the high- and -low-food groups (Fig. 9D; Mann–Whitney *U* = 16.0, *P* = 0.277, two tailed, α = 0.013).

For crabs in the high-food group, there was no significant difference in final BPT (Mann–Whitney *U* = 12.0, *P* = 0.064, two tailed, α = 0.013) or FLT (Mann–Whitney *U* = 37.0, *P* = 0.298, two tailed, α = 0.013) between the summer and winter feeding trials. For crabs in the low-food group, there was also no significant difference in final BPT (Mann–Whitney *U* = 11.0, *P* = 0.030, two-tailed, α = 0.013) or FLT (Mann–Whitney *U* = 36.0, *P* = 0.634, two-tailed, α = 0.013) between the summer and winter feeding trials.

## Discussion

In the present study, we investigated the effects of food limitation on growth, oxygen consumption rates (${\dot{M}}_{O_{2}}$), temperature sensitivity and upper temperature tolerance of juvenile Dungeness crabs from the SFE to determine how changes in food availability might impact physiological trade-offs between growth and stress tolerance. We hypothesized that food limitation would negatively affect energy supply (measured as ${\dot{M}}_{O_{2}}$ and aerobic capacity of two metabolic enzymes) and that crabs would compensate by reducing the energy allocation to aerobic scope functions (measured as growth) while maintaining energy supply to basal maintenance functions, such as circulation (measured as heart rate) and cellular homeostasis, to preserve acute tolerance to environmental stressors, such as high temperature (measured as upper -thermal -tolerance). Overall our results demonstrated that while food limitation negatively affected growth of juvenile Dungeness crabs in both the summer and winter feeding trials, crabs in the low-food group maintained both ${\dot{M}}_{O_{2}}$ at ambient SFE temperatures (15°C, summer trial only) and upper temperature tolerance as determined by failure of cardiac function when compared with crabs in the high-food group (summer and winter trials). Providing evidence of a physiological trade-off, food-limited crabs appear to allocate energy away from growth and towards mechanisms that preserve stress tolerance limits during a period of rapid growth and development. It is of note, however, that during acute exposures to increased temperature, food-limited crabs were unable to increase their ${\dot{M}}_{O_{2}}$ to the same level as that achieved by well-fed crabs and therefore, if exposure to elevated temperatures persists and requires more energy than can be met by crabs in their food-limited state, physiological performance may be compromised.

### Effects of food limitation on growth

Growth and moulting are energy-intensive processes. If a crab's energy reserves are low due to food limitation, it may divert energy away from growth to preserve other facets of basal maintenance, such as ion regulation, acid–base regulation, protein turnover, ventilation, circulation or excretion (Terwilliger and Dumler, 2001; Sokolova *et al.*, 2012). In both the summer and the winter feeding trials, crabs in the low-food group gained, on average, only 40–50% of the weight of crabs in the high-food group (Fig. 1), which supports findings from previous studies showing that food limitation reduces growth (Mayrand *et al.*, 2000; Wen *et al.*, 2006). During the summer feeding trial, food limitation also decreased moulting frequency (Fig. 2) and the growth increment following moulting (Fig. 3), consistent with other studies of moulting in crustaceans (Chittleborough, 1975; Terwilliger and Dumler, 2001; Wen *et al.*, 2006). During the winter feeding trial, there was no significant difference in moulting between feeding groups (Fig. 4); however, moulting frequency was much lower in the winter compared with the summer. A similar trend was observed, with greater moulting in the high- compared with the low-food group during the last two-thirds of the feeding trial. It is likely that crabs in the low-food group had sufficient energy stores accumulated from feeding in the SFE prior to experimentation; however, after 2 weeks, these stores were depleted, and the impacts of food limitation on different growth parameters became more apparent. The results from this study demonstrate that food limitation resulted in lower growth rates, with more pronounced effects in crabs from the summer feeding trial, and suggest that juvenile Dungeness crabs, during natural periods of rapid growth, allocate less energy to growth when food becomes limited.

### Effects of food limitation on aerobic -metabolism

Although crabs in the low-food group slowed their growth in response to food limitation, our results suggest that it is not a result of reduced energy supply because food-limited crabs maintained similar values of ${\dot{M}}_{O_{2}}$, one of our metrics of ATP supply via aerobic metabolism, at 15°C to crabs in the high-food group (Fig. 5). Crabs in the low-food group were either consuming enough energy from the food ration provided to balance energy demands or, more likely, had enough energy stored as reserves in order to preserve energy supply to basal maintenance functions as seen in Southern King crabs (*Lithodes santolla*; Comoglio *et al.*, 2008) and in False Southern King crabs (*Paralomis granulosa*; Comoglio *et al.*, 2005). It is well known that ${\dot{M}}_{O_{2}}$ increases after feeding in crustaceans (Houlihan *et al.*, 1990; Whiteley *et al.*, 2001; Curtis and McGaw, 2010; McGaw and Whiteley, 2012) and therefore, crabs in the high-food group may have had elevated values of ${\dot{M}}_{O_{2}}$ as a result of being fed more recently (i.e. 24–48 h before trials compared with multiple days in the low-food group). Further research is needed in order to gain a better understanding of specific dynamic action and gastric processing in juvenile Dungeness crabs. Our results suggest that digestion may not pose additional metabolic demands for long periods of time in juvenile Dungeness crabs because the values of ${\dot{M}}_{O_{2}}$ of crabs from the high-food group at 15°C, our control temperature, were not significantly elevated above the ${\dot{M}}_{O_{2}}$ of crabs from the low-food group. Given that juvenile Dungeness crabs in the low-food group experienced reductions in growth but no reductions in ${\dot{M}}_{O_{2}}$ at 15°C, and assuming that the crabs maintained a balance between energy supply and demand, energy was probably diverted away from growth and towards increased basal maintenance demands or an aspect of defense against stress associated with food limitation (Sokolova *et al.*, 2012). Additional studies are needed to understand where energy may have been reallocated in crabs from the low-food group and how much longer crabs could have persisted in the food-limited state before ${\dot{M}}_{O_{2}}$ would need to be suppressed.

Some insight into where energy may have been allocated can be gleaned from the biochemical analyses of the metabolic enzymes CS and MDH. While there was no effect of food limitation on whole-body aerobic metabolism at 15°C, increased CS specific activity in gill tissue of crabs in the low-food group suggested a higher branchial aerobic metabolic capacity compared with crabs in the high-food group (Fig. 6). In fish, food limitation has been shown to cause catabolism of fat stores, and this oxidative metabolism was a source of reactive oxygen species (Morales *et al.*, 2004), leading to oxidative stress in gill (Bayir *et al.*, 2011) and liver tissues (Pascual *et al.*, 2003; Morales *et al.*, 2004). Food limitation in the present study may also have resulted in oxidative stress in gill tissues, and increased CS specific activity was an indicator of elevated basal maintenance costs in gill tissues to upregulate antioxidant defense and oxidative stress repair mechanisms. Gill -tissue ${\dot{M}}_{O_{2}}$ and biochemical assays of oxidative stress and antioxidant defense would be required to explore this hypothesis further.

Despite differences in CS specific activity, MDH specific activity was similar in the gill tissues of crabs from the low- and high-food groups (Fig. 7). A greater ratio of MDH to CS specific activity in crabs from the high-food group suggests that MDH may have had an additional role in crabs from the high-food group. In addition to being part of the citric acid cycle, MDH is also involved in gluconeogenesis and anaerobic metabolism (Minarik *et al.*, 2002). Crabs in both food groups had similar values of ${\dot{M}}_{O_{2}}$ at 15°C, so crabs from the high-food group were unlikely to be oxygen limited and reliant on anaerobic metabolism. It is more likely that MDH in crabs from the high-food group was used for increased gluconeogenesis and storage of energy reserves, although storage is not localized to the gills. In both the summer and the winter feeding trials, crabs in the high-food group gained significantly more weight than crabs in the low-food group, supporting the idea that crabs in the high-food group allocated more energy towards storage (Fig. 1). Additional studies are needed to measure the effects of food limitation on glucose and glycogen levels in multiple tissues (Lim and Ip, 1989; Auerswald *et al.*, 2009) in juvenile Dungeness crabs in order to understand whether gluconeogenesis could explain differences in the ratio of MDH to CS specific activity between crabs in the high- and low-food groups.

When faced with elevated temperatures, crabs in both the low- and the high-food groups increased their ${\dot{M}}_{O_{2}}$ due to the physiocochemical thermal sensitivity of metabolism, which is consistent with previous studies of Dungeness crabs (Prentice and Schneider, 1979; Gutermuth and Armstrong, 1989; Brown and Terwilliger, 1999); however, crabs in the low-food group did not increase ${\dot{M}}_{O_{2}}$ to the same degree as crabs in the high-food group (Fig. 5). A study of the tropical freshwater crab *Paratelphusa hydrodromus* found that starvation caused reductions in oxygen consumption at ambient temperature and during thermal stress (Kotaiah and Rajabai, 1975) due to decreases in energy demand associated with reduced energy supply. In the present study, food-limited juvenile Dungeness crabs may have had enough energy in their energy reserve pools to maintain aerobic metabolism at 15°C, but lacked sufficient reserve pools to match the energy demand imposed by elevated temperatures, which was matched in crabs from the high-food group during thermal stress.

### Effects of food limitation on cardiac -performance

Numerous studies have demonstrated that food limitation affects the physiological response of crabs to environmental stressors (Whiteley *et al.*, 2001; McGaw, 2005; McGaw and Whiteley, 2012) and temperature sensitivity (Curtis and McGaw, 2012). In the present study, there were no differences in the heart rates of crabs in the high- and low-food groups at 15°C (Fig. 8), suggesting that food limitation did not affect cardiac performance at current SFE water temperatures and that energy allocation to the basal circulation was maintained during both summer and winter feeding trials. As temperature increased, heart rates of crabs in the low-food group kept pace with those of crabs in the high-food group at all but one temperature (33°C) during the summer feeding trial. Although crabs in the low-food group were unable to increase ${\dot{M}}_{O_{2}}$ at elevated temperatures to the same degree as crabs in the high-food group, food-limited crabs appeared to have allocated energy to maintain cardiac performance and haemolymph circulation, an important aspect of basal maintenance. As temperatures approached the upper critical thermal limits of cardiac performance, only slight impairment in heart function was observed in food-limited crabs, although not significantly. In addition to maintaining heart rate during thermal stress, crabs from the low-food group maintained the same upper thermal tolerance, assessed by both final BPT and FLT, as crabs in the high-food group (Fig. 9); therefore, while crabs in the low-food group had lower values of ${\dot{M}}_{O_{2}}$ at high temperatures, energy was probably allocated to conserve an upper thermal tolerance similar to that of crabs in the high-food group. While some studies have found a negative correlation between food limitation and upper thermal tolerance (Woiwode and Adelman, 1992; Nyamukondiwa and Terblanche, 2009; Woodman, 2010), the lack of an effect of food limitation on the temperature at which heart function failed (BPT) or ceased (FLT) in juvenile Dungeness crabs, coupled with the growth reductions observed during the feeding trials, suggests that crabs in the low-food group allocated energy away from growth and towards acute and rapid cellular stress response mechanisms (Kültz, 2005).

The effects of food limitation on cardiac performance varied between seasons. Given that juvenile crabs are undergoing a rapid period of growth while they reside in the SFE, it is not possible to separate the effects of season from developmental stage. The maximal heart rate achieved by crabs in the winter was significantly lower than that of crabs in the summer, possibly due to the negative relationship between body size and heart rate (Ahsanull and Newell, 1971; Aagaard, 1996; Eshky *et al.*, 1996; Spicer, 2001) and the larger size of crabs in the winter feeding trial or due to the slightly reduced water temperatures (by ~3°C) in the winter trial (Styrishave *et al.*, 1999; Camacho *et al.*, 2006). In contrast to documented seasonal differences in the upper temperature tolerance of other crustaceans, there was no difference in the upper thermal tolerance between crabs in the summer and winter feeding trials (Cuculescu *et al.*, 1998; Stillman and Somero, 2000; Camacho *et al.*, 2006). Previous studies have acclimated crustaceans to much larger differences in temperature across seasons (i.e. 10–16 vs. ~3°C in the present study). While it is possible that acclimation temperature may impact the critical upper thermal tolerance of juvenile Dungeness crabs, the relatively small differences in seasonal temperatures in the SFE may allow crabs to maintain a high upper temperature tolerance throughout their residence time on the nursery grounds in order to cope with the highly variable nature of estuarine environments.

### Conclusion

Juvenile Dungeness crabs living in food-limited habitats are likely to be able to maintain aerobic metabolism at current estuarine temperatures; however, energy supply (measured by ${\dot{M}}_{O_{2}}$), as temperatures are increased, may decrease despite the ability of the crabs to maintain upper temperature tolerance across summer and winter seasons. It appears, however, that the ability to maintain basal maintenance functions and tolerate thermal stress when food is limited comes at a physiological trade-off to growth. Crabs that inhabit food-limited habitats in the SFE may grow more slowly, and there could be consequences associated with smaller body size and slower growth, including an increased likelihood of predation or an increased period of estuary residence required to reach outmigration size. While food limitation may cause reductions in growth rate, the fact that food-limited crabs maintained upper temperature tolerance suggests that the juvenile Dungeness crab populations using the SFE as a nursery may not be particularly vulnerable to short-term foraging in high-temperature environments or to an increased frequency of acute thermal extremes predicted to occur in the next century (Hayhoe *et al.*, 2004; Cloern *et al.*, 2011; Wagner *et al.*, 2011). Additional research is needed to understand the combined impacts of acclimation to warmer aquatic environments and reduced food availability on temperature sensitivity and upper temperature tolerance. More chronic exposure to elevated temperature would impact energy demand because biochemical reaction rates are increased and could exacerbate the effects of limited energy supply on energy allocation in food-restricted environments.
